# The Heat Shock Protein 90 (HSP90) Is Required for the IL-33-Induced Cytokine Production in Mast Cells (MCs)

**DOI:** 10.3390/ijms231810855

**Published:** 2022-09-17

**Authors:** Isabel Peters, Sylvia Müller, Claudia Küchler, Ute Jäger, Sebastian Drube

**Affiliations:** Institut of Immunology, Universitätsklinikum Jena, Friedrich-Schiller-Universität Jena, Leutragraben 3, 07743 Jena, Germany

**Keywords:** mast cells, IL-33, HSP90, 17AAG

## Abstract

The alarmin interleukin-33 (IL-33) is released upon cell stress and damage in peripheral tissues. The receptor for IL-33 is the Toll/Interleukin-1 receptor (TIR)-family member T1/ST2 (the IL-33R), which is highly and constitutively expressed on MCs. The sensing of IL-33 by MCs induces the MyD88−TAK1−IKK2-dependent activation of p65/RelA and MAP-kinases, which mediate the production of pro-inflammatory cytokines and amplify FcεRI-mediated MC-effector functions and the resulting allergic reactions. Therefore, the investigation of IL-33-induced signaling is of interest for developing therapeutic interventions effective against allergic reactions. Importantly, beside the release of IL-33, heat shock proteins (HSPs) are upregulated during allergic reactions. This maintains the biological functions of signaling molecules and/or cytokines but unfortunately also strengthens the severity of inflammatory reactions. Here, we demonstrate that HSP90 does not support the IL-33-induced and MyD88−TAK1−IKK2-dependent activation of p65/RelA and of mitogen-activated protein (MAP)-kinases. We found that HSP90 acts downstream of these signaling pathways, mediates the stability of produced cytokine mRNAs, and therefore facilitates the resulting cytokine production. These data show that IL-33 enables MCs to perform an effective cytokine production by the upregulation of HSP90. Consequently, HSP90 might be an attractive therapeutic target for blocking IL-33-mediated inflammatory reactions.

## 1. Introduction

IL-33 is a central component that directly and/or indirectly induces the activation of cells of the innate and the adaptive immune system. Thereby, IL-33 is constitutively expressed in endothelial and epithelial cells [1]. Cell damage by environmental triggers (e.g., allergens) results in a passive release of IL-33 [1,2]. Liberated IL-33 is sensed by MCs, which highly and constitutively express the IL-33R. Therefore, MCs sense cellular damage in peripheral tissues by recognizing alarmins such as IL-33 [2]. Like all members of the IL-1 cytokine family, IL-33, via the IL-33R, induces the MyD88−TAK1−IKK2-dependent activation of p65/RelA and of MAP-kinases, which results in a pro-inflammatory cytokine response [1,3]. This culminates in the recruitment of neutrophils and monocytes into peripheral tissues and finally initiates allergic reactions such as contact hypersensitivity reactions (CHS) independently from the adaptive immune system [4,5,6]. IL-33 also supports MC-dependent type-I hypersensitivity reactions [7]. Thereby, the immunoglobulin E (IgE)/FcεRI-mediated nuclear factor of activated T-cells (NFAT) activation in MCs is synergistically increased by IL-33 and thus leads to a boosted cytokine response and allergic inflammation [7,8].

Allergic reactions are mediated by HSPs [9,10]. HSPs are ubiquitously expressed and highly conserved intracellular proteins categorized into families depending on their molecular weight into HSP110, HSP90, HSP70, HSP60, HSP27, and small HSPs [11]. Reflecting their important role in allergic reactions, HSPs are upregulated in response to cellular stress to maintain the biological function of signaling molecules and cytokines in cells of the innate and adaptive immune system [11]. HSP90 is a predominantly expressed HSP and acts as a regulator of p65/RelA by controlling the stability and therefore the activation of the TAK1/IKK2 module in cells stimulated with TNFα, IL-1β, or phorbol 12-myristate 13-acetate (PMA) [12,13,14]. To block HSP90 activity, several drugs have been developed such as 17-allylamino-17-demethoxy-geldanamycin (17-AAG), which binds to the N-terminal ATP/ADP-binding pocket of HSP90 [15]. 17AAG-mediated HSP90 inactivation results in the destabilization and degradation of HSP90 client proteins [16]. Given that HSP90 mediates the stability and activation of the TAK1/IKK2 module [12,13,14], 17AAG and related drugs have been proven as attractive targets for treating inflammatory responses. The role of HSP90 in MCs is poorly understood. Here, we aimed to characterize the role of HSP90 in IL-33-activated MCs by using the highly selective HSP90 inhibitor 17AAG.

## 2. Results

### 2.1. IL-33 Upregulates HSP90

**Figure 1:**
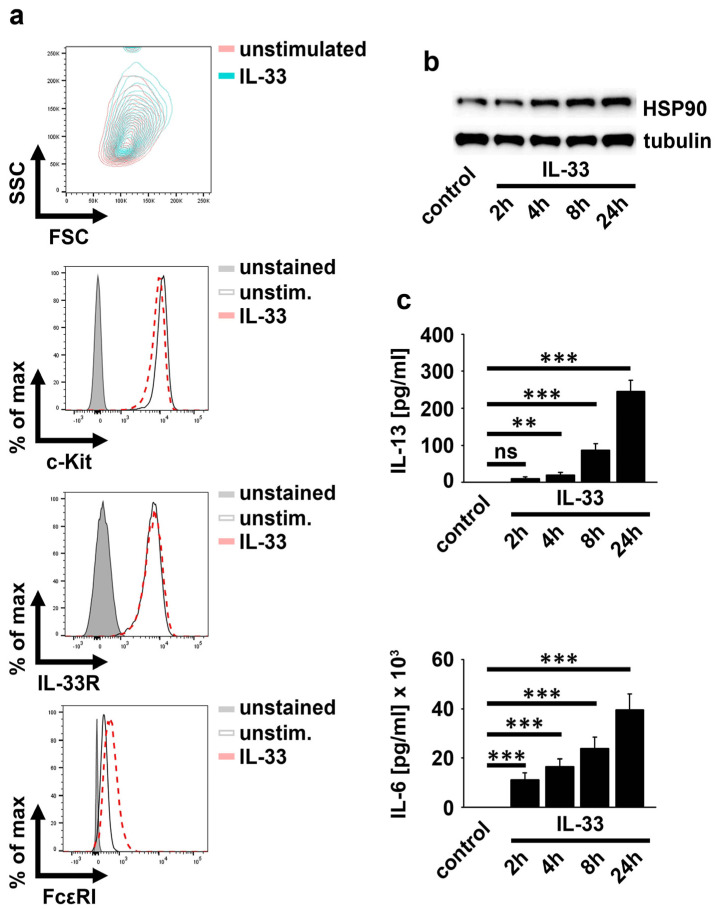
IL-33 upregulates HSP90 expression. (**a**) BMMCs were stimulated with IL-33 (50 ng/mL) for 24 h. Afterwards, cells were analyzed by flow cytometry (one representative experiment is shown). (**b**,**c**) BMMCs were stimulated with IL-33 (50 ng/mL) as indicated. Cells were lysed and analyzed by Western blotting (one representative Western blot experiment is shown), (**b**) or the supernatants were collected and analyzed by ELISA (**c**) (the summary of *n* = 4 biological replicates is shown; unpaired Student’s *t*-test; ns: not significant, ** *p* ≤ 0.01, *** *p* ≤ 0.001).

The expression and the release of IL-33 and HSP90 are upregulated in psoriasis patients [10]. Given the presence of MCs in peripheral tissues, we speculated a functional link between released IL-33 and HSP90 in MCs. Therefore, we aimed to investigate the impact of HSP90 on IL-33-induced MC-effector functions. IL-33 at concentrations between 10 ng/mL and 100 ng/mL induced maximal effector functions in MCs [17]. Therefore, we decided to use 50 ng/mL IL-33 for all the experiments. As shown in Figure 1a, IL-33 slightly increased the MC granularity but did not affect the surface expression of c-Kit and the IL-33R. In contrast to this, the FcεRI was upregulated by IL-33, indicating the sensitization of MCs for IgE stimulation. Notably, IL-33 upregulated HSP90, which correlated with the increased cytokine production (Figure 1b,c). This indicated a functional link between upregulated HSP90 and altered MC characteristics as well as the increased cytokine response upon IL-33 stimulation.

### 2.2. IL-33 Requires HSP90 to Mediate the Production of Cytokines in MCs

**Figure 2:**
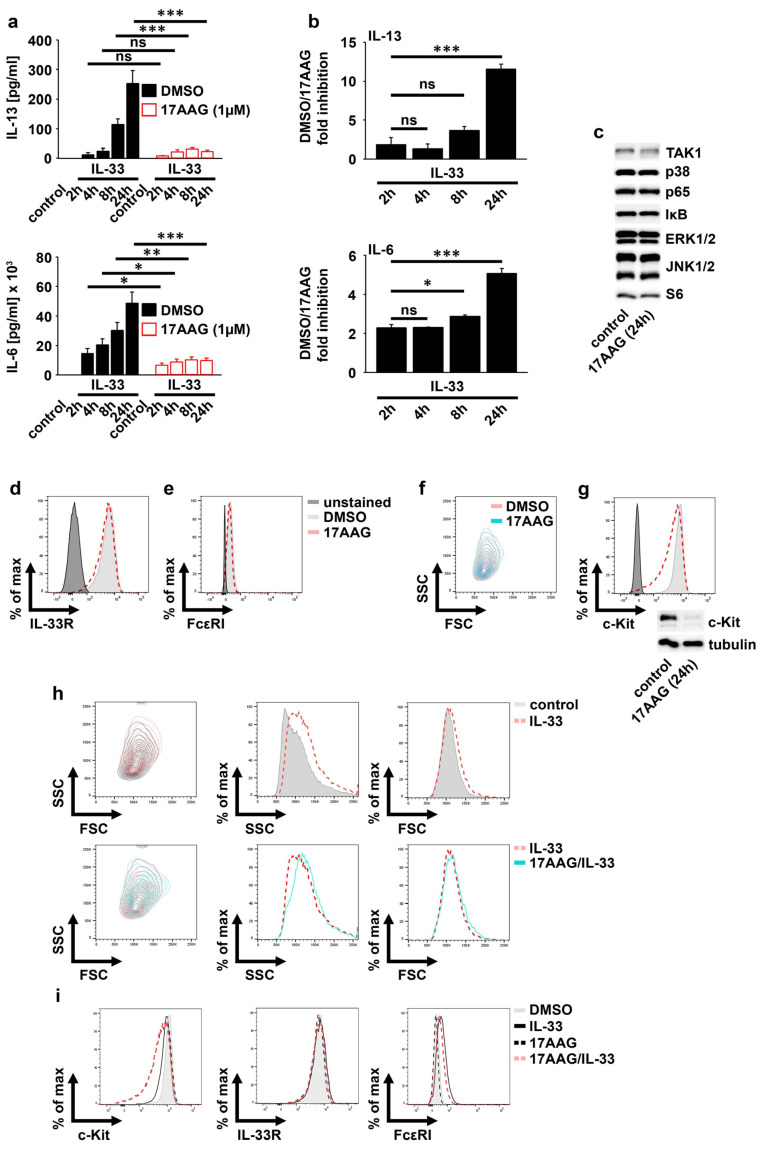
HSP90 mediates IL-33-induced cytokine production. (**a**) BMMCs treated with DMSO (vehicle) or 17AAG (1 µM) were stimulated with IL-33 (50 ng/mL) as indicated. Supernatants were collected and analyzed by ELISA (the summary of *n* = 3 biological replicates is shown; unpaired Student’s *t*-test; ns: not significant, * *p* ≤ 0.05, ** *p* ≤ 0.01, *** *p* ≤ 0.001). (**b**) The fold inhibition of the produced cytokines in the presence of 17AAG is shown. The concentrations of the produced cytokines in vehicle- or 17AAG-treated and IL-33-stimulated BMMCs as shown in (**a**) were used to calculate the DMSO−IL-33/17AAG−IL-33 rate (the summary of *n* = 3 biological replicates is shown; unpaired Student’s *t*-test; ns: not significant, * *p* ≤ 0.05, *** *p* ≤ 0.001). (**c**) BMMCs were treated with 17AAG (1 µM) for 24 h. BMMCs were lysed and analyzed by Western blotting. (**d**–**g**) BMMCs were treated with 17AAG (1 µM) for 24 h. Afterwards, cells were analyzed by flow cytometry (one representative experiment is shown) or were lysed and analyzed by Western blotting (one representative experiment is shown) (**g**). (**h**,**i**) BMMCs were treated with DMSO (vehicle) or 17AAG (1 µM) and were stimulated with IL-33 (50 ng/mL). After 24 h, cells were analyzed by flow cytometry (one representative experiment is shown).

Given that IL-33 upregulates the expression of HSP90, we investigated whether IL-33-induced effector functions depend on HSP90 by using the HSP90-specific inhibitor 17AAG. First, we titrated the concentration of 17AAG to exclude cytotoxic side effects of 17AAG in our functional assays. In contrast to 0.5 µM and 1 µM, 2 µM 17AAG induced cell death in MCs (Figure S1a). Furthermore, compared to 0.5 µM, 1 µM 17AAG reduced the strong IL-33-induced IL-6 production more efficiently (Figure S1b). Consequently, we used 1 µM 17AAG. As shown in Figure 2a, the IL-33-induced and time-dependent increase in IL-6 and IL-13 was reduced by 17AAG. Thereby, the inhibitory effect of 17AAG was more pronounced in MCs stimulated for 24 h with IL-33 (Figure 2b), which was also shown in ex vivo-expanded peritoneal-cavity MCs (PCMCs) (Figure S1c). This demonstrated that HSP90 supports the cytokine production induced by short-term IL-33 stimulations, while it is essentially required to mediate an effective cytokine response in MCs subjected to long-term IL-33 stimulations. HSP90 stabilizes proteins by maintaining their active conformation [13]. Therefore, HSP inhibition results in the degradation of client proteins such as TAK1 or c-Kit [13]. We speculated that 17AAG alone destabilized proteins that are necessary for IL-33-induced signaling. However, TAK1, p38, p65/RelA, ERK1/2, JNK1/2, and S6 were stable in the presence of 17AAG (Figure 2c). Furthermore, in unstimulated MCs, 17AAG also did not affect the surface expression of the IL-33R (Figure 2d) or FcεRI (Figure 2e) as well as the MC granularity (Figure 2f). In contrast to this, the surface expression as well as the total expression of c-Kit was strongly reduced by 17AAG in unstimulated MCs (Figure 2g). 

We found that IL-33 increased the MC granularity and the expression of the FcεRI. However, confirming the data from unstimulated BMMCs, 17AAG also did not influence the upregulation of MC granularity and of the FcεRI surface expression induced by IL-33 (Figure 2h,i). These data demonstrated that HSP90 is essential for IL-33-induced cytokine production and stabilization of c-Kit on MCs but is dispensable for MC granularity and the surface expression of the IL-33R and the FcεRI.

### 2.3. HSP90 Is Not Required for IL-33-Induced p65/RelA Activation

**Figure 3:**
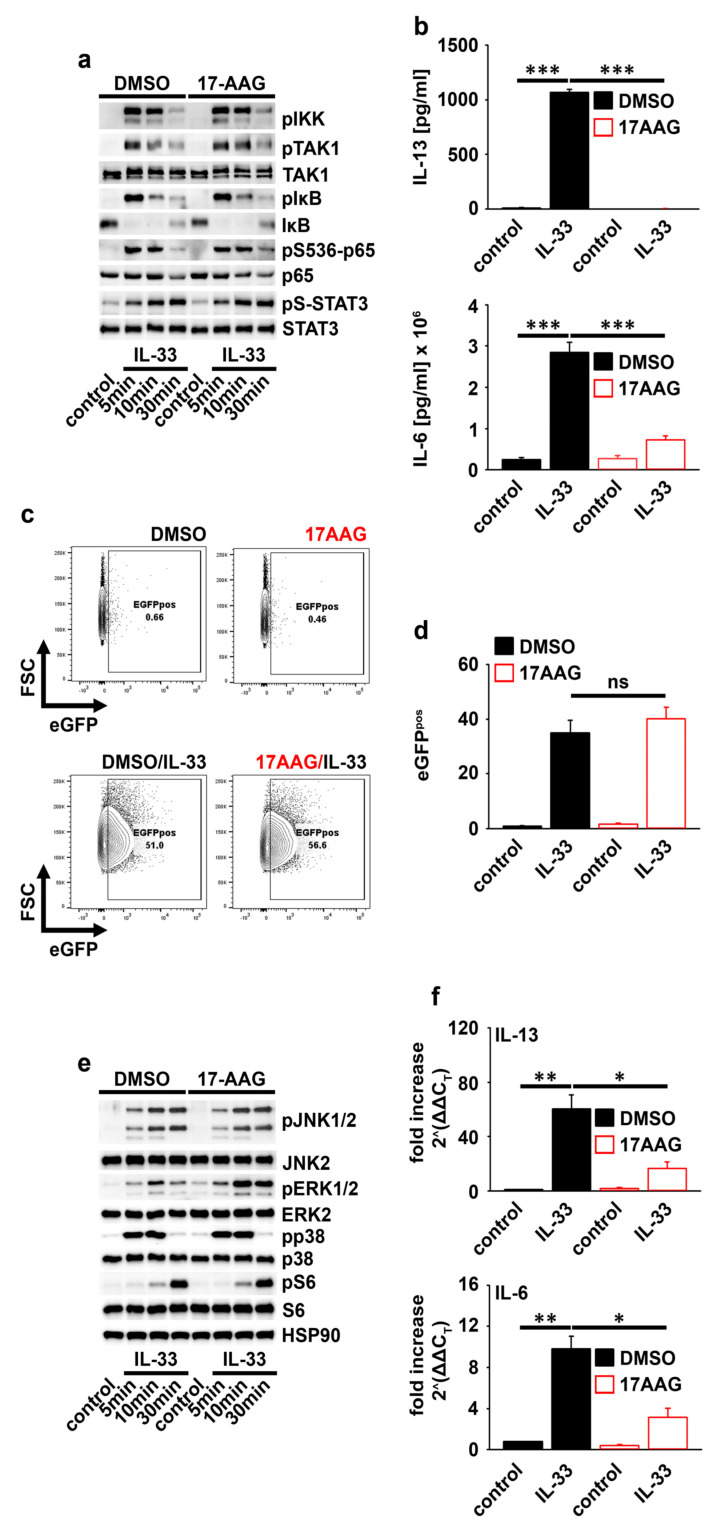
HSP90 mediates cytokine transcript stability. (**a**) BMMCs treated with DMSO (vehicle) or 17AAG (1 µM) were stimulated with IL-33 (50 ng/mL) as indicated. Cells were lysed and analyzed by Western blotting (one representative Western blot experiment is shown). (**b**) P65/RelA−eGFP MC/9 cells were treated with DMSO (vehicle) or 17AAG (1 µM) and were stimulated with IL-33 (50 ng/mL) for 24 h. Supernatants were collected and analyzed by ELISA (the summary of *n* = 3 replicates is shown; unpaired Student’s *t*−test; *** *p* ≤ 0.001). (**c**) P65/RelA−eGFP MC/9 cells were treated with DMSO (vehicle) or 17AAG (1 µM) and were stimulated with IL-33 (50 ng/mL) for 24 h. Cells were harvested and analyzed for eGFP expression by flow cytometry (one representative experiment is shown). (**d**) Shown is the statistical analysis of the experiment shown in (**c**) (the summary of *n* = 5 replicates is shown; unpaired Student’s *t*-test; ns: not significant). (**e**) BMMCs were treated with DMSO (vehicle) or 17AAG (1 µM) and were stimulated with IL-33 (50 ng/mL) as indicated. Cells were lysed and analyzed by Western blotting (one representative Western blot experiment is shown). (**f**) BMMCs were treated with DMSO (vehicle) or 17AAG (1 µM) and were stimulated with IL-33 (50 ng/mL) for 6 h. Cells were harvested and analyzed by qPCR (the summary of *n* = 3 biological replicates is shown; unpaired Student’s *t*−test; * *p* ≤ 0.05, ** *p* ≤ 0.01).

IL-33 induces p65/RelA and MAP-kinase activation [4,18,19]. 17AAG influenced neither the IL-33-induced activation of TAK1 and IKK2 nor the phosphorylation of p65/RelA (Figure 3a). The p65/RelA phosphorylation did not necessarily correlate with the p65/RelA activity. Therefore, we used the p65/RelA reporter MC line p65/RelA−eGFP MC/9. In this MC line, the p65/RelA binding side is located upstream of the eGFP cassette. Thus, the binding of p65/RelA leads to eGFP expression, which is detectable by flow cytometry and indicates p65/RelA activation. As shown for primary MCs, the IL-33-induced cytokine response was also blocked by 17AAG in the p65/RelA−eGFP MC/9 mast cell line (Figure 3b). However, 17AAG did not affect the IL-33-induced eGFP production (Figure 3c,d). Together these data demonstrated that the IL-33-induced p65/RelA activation is mediated independently from HSP90.

### 2.4. HSP90 Negatively Regulates the Activation of MEK1/2−ERK1/2 Signaling

IL-33 activates p38, ERK1/2, and JNK1/2 [4]. Thereby, p38 activation is essential for IL-33-induced cytokine production [4]. Furthermore, Koga et al. found that HSP90 inhibition activates ERK1/2 [20]. Given these facts, we determined the role of HSP90 in IL-33-induced MAP-kinase signaling. Confirming the regulatory role of HSP90 in ERK1/2 activation [20] and thus demonstrating the functionality of 17AAG, the IL-33-induced ERK1/2 activity was selectively enhanced. However, p38, JNK1/2, and the p70S6K-S6 module remained unchanged (Figure 3e). Given that the IL-33-induced cytokine response was inhibited by 17AAG, these data show that MAP-kinases are not involved in HSP90-mediated cytokine production. In summary, the HSP90-dependent cytokine production is mediated downstream of the IL-33-induced p65/RelA and MAP-kinase activation.

### 2.5. HSP90 Stabilizes the IL-6 and IL-13 Transcripts

HSP90 is not required for the IL-33-induced p65/RelA and MAP-kinase activation. Therefore, we speculated that HSP90 is important for the stability of cytokine mRNAs. Indeed, 17AAG strongly decreased the transcript levels of IL-6 and IL-13 produced in response to IL-33 in MCs (Figure 3f). These data showed that HSP90 is important for the stability of cytokine mRNAs.

### 2.6. HSP90 Is Also Essential for the SCF-Supported and IL-33-Induced Cytokine Production in MCs

The c-Kit ligand, the stem cell factor (SCF), supports IL-33-induced MC-effector functions, resulting in a potentiated cytokine production [21,22]. Due to downregulated c-Kit, we tested the functionality of activated c-Kit in the presence of 17AAG regarding IL-33-induced MC-effector functions. Compared to either stimulus alone, co-stimulation with SCF and IL-33 more efficiently increased MC granularity, upregulated the surface expression of the FcεRI, and potentiated the induced cytokine production (Figure S2a–c). Except for the SCF/IL-33-induced cytokine production, neither the increased granularity nor upregulated FcεRI (Figure S2a–c) were sensitive to 17AAG treatment. Next, we determined the influence of SCF on IL-33-induced p65/RelA activation. Thus, we again used the p65/RelA−eGFP MC/9 reporter MC line. Consistent with the results for primary MCs, co-stimulation with SCF and IL-33 also induced a potentiated and 17AAG-sensitve cytokine production in the p65/RelA−eGFP MC/9 reporter MC line (Figure S3a). However, the IL-33-induced p65/RelA activation in this MC line was only slightly increased by SCF and was insensitive to 17AAG (Figure S3b). In contrast to this, the stability of the strongly increased IL-6 and IL-13 transcript levels was reduced in the presence of 17AAG (Figure S3c). In line with single IL-33 stimulations, these results demonstrate that HSP90 is required for the SCF/IL-33-induced potentiated cytokine production but not the upregulated granularity or the altered receptor surface expression in MCs.

## 3. Discussion

IL-33 and HSP90 are upregulated in psoriasis patients [10]. The release of both, IL-33 and HSP90 mediates inflammatory responses [5,7,9,10]. In this manuscript, we identified a link between IL-33 sensing and HSP90 in MCs. We showed that IL-33 upregulates HSP90, which results in an HSP90-dependent cytokine response. HSP90 stabilizes cell surface receptors and intracellular signaling molecules such as the TAK1/IKK2 signaling module [12,13]. However, we found that neither the stability of the IL-33R nor downstream signaling molecules are dependent on HSP90 in MCs. Exclusively, ERK1/2 activation was enhanced in response to HSP90 inhibition. HSP90 maintains the inactive conformation of SFKs [20,23]. Therefore, the inhibition of HSP90 activates the Src−ERK1/2 module, which results in an enhanced ERK1/2 activation [20,23]. However, given the prominent role of p65/RelA and p38 in IL-33-induced effector functions [4,19,24], the 17AAG-caused increased ERK1/2 activation is not sufficient to influence the IL-33-induced cytokine production in MCs. These data demonstrated that HSP90 did not substantially influence the IL-33-induced signaling. However, we found that HSP90 acts downstream of the IL-33-induced signaling and mediates the stability of cytokine mRNAs that are produced in response to the IL-33-induced p65/RelA and p38 activation [4,18,19]. The fact that the IL-33-induced cytokine production but not the production of eGFP was blocked by HSP90 inactivation in p65/RelA−eGFP MC/9 cells showed the differential regulation of eGFP and cytokine transcripts. This is in line with the HSP90-independent increased MC granularity and the increased surface expression of the FcεRI in response to IL-33 stimulation, which were not dependent on HSP90. Consequently, we conclude that HSP90 selectively mediates the stability of cytokine transcripts but not of transcripts encoding proteins that are involved in the upregulation of MC granularity or in the increased surface expression of the FcεRI. Importantly, cytokine transcripts contain adenine-/uridine-rich elements (AREs), which control the stability of mRNAs [25]. These cis elements recruit ARE-binding proteins that regulate whether transcripts are translated or degraded [25]. Due to the strongly reduced production of cytokine transcripts in the presence of 17AAG, we suggest that HSP90 mediates the stability of ARE-binding proteins and/or their association with the cytokine transcripts. Recently, HSP90 and IL-33 were shown to be upregulated in psoriasis patients [9,10]. In line with that, in animal models, HSPs [26,27] and IL-33-activated MCs were identified as import for the development of allergic reactions such as contact hypersensitivity (CHS) [4,5]. Here, we identified a functional link between HSP90 and the IL-33-induced cytokine response in MCs. Thereby, HSP90 is not relevant for the IL-33R−TAK1−IKK2−p65/RelA/p38 signaling axis but mediates the cytokine transcript stability. Together, these data demonstrate that HSP90 in MCs is essential for mediating IL-33-driven allergic reactions.

## 4. Materials and Methods

### 4.1. Mice

Wild-type (WT) mice (C57/BL6J) were maintained at the Animal Research Facility of the Jena University Hospital. We used sex- and age-matched wild-type (wt) mice. The isolation of murine bone marrow was approved by the Thüringer Landesamt für Lebensmittelsicherheit und Verbraucherschutz (TLL−V), Bad Langensalza. The organ isolation license for the Institute of Immunology, Jena, is twz−36−2017.

### 4.2. BMMC Generation

Bone marrow of wt mice was cultured in IMDM (Gibco) (with 10% FCS, 100 U/mL penicillin, 100 mg/mL streptomycin, 50 mM β−mercaptoethanol, and 20 ng/mL X63Ag-653 BPV−rmIL-3 supernatant, the source of rmIL-3 for BMMC generation). Until the 5th week in culture, the medium was replaced with fresh medium every 2nd day. From the 5th week, the medium was changed two times a week. The purity of the BMMCs was determined by the surface expression of c-Kit, FcεRI, and T1/ST2 by flow cytometry. When the cell cultures consisted of 95% BMMCs, the cell cultures were used for experiments for 4–5 weeks.

### 4.3. Expansion of Peritoneal-Cavity-Derived MCs (PCMCs)

Peritoneal-cavity cells were isolated by flushing the peritoneal cavity with ice-cold PBS. One biological sample consisted of peritoneal-cavity cells from 5 mice. For *n* = 3 biological replicates, 15 wt mice were used. To expand the PCMCs, the cells were cultured in IMDM (Gibco) supplemented with 10% FCS, 100 U/mL penicillin, 100 µg/mL streptomycin, 50 mM β-mercaptoethanol, rmSCF (10 ng/mL), and rmIL-3 (10 ng/mL) (Peprotech, Cambridge, UK) for 14–21 days. To analyze the purity of the PCMCs, we determined the percentage of c-Kit^+^/FcεRI^+^/T1/ST2^+^ cells in the cultures (on day 14 or day 21) by flow cytometry. Cultures with a purity of 90–95% c-Kit^+^/FcεRI^+^/T1/ST2^+^ (PCMC) cells were used for further experiments.

### 4.4. Culture of p65/RelA-eGFP MC/9 Reporter MC Line [28]

P65/RelA-eGFP MC/9 reporter cells were cultured in RPMI (Sigma, St. Louis, MO, USA) (supplemented with 10% FCS, 100 U/mL penicillin, 100 mg/mL streptomycin, 50 mM β-mercaptoethanol, and 20 ng/mL X63Ag-653 BPV-rmIL-3 supernatant, the source of rmIL-3).

### 4.5. Stimulation of MCs (BMMCs, PCMCs, or p65/RelA−eGFP MC/9 Cells)

BMMCs, PCMCs, or the p65/RelA-eGFP MC/9 reporter MCs were washed to remove IL-3 and were seeded in IL-3-free media at a density of 1 × 10^6^ cells/mL. After 1 h, the cells were treated with vehicle (DMSO) or the HSP90 inhibitor 17AAG (the concentrations are indicated in the figures) for 30 min. Subsequently, the cells were stimulated with rmSCF and/or rmIL-33 (50 ng/mL) (Peprotech).

### 4.6. Flow Cytometry

After stimulation with rmIL-33 and/or rmSCF (both 50 ng/mL) (Peprotec), the BMMCs were washed with PBA (PBS supplemented with 5 mg/mL BSA and 10 mM NaN_3_). Afterwards, we added anti−CD16/CD32 and rat-IgG (Jackson) to prevent non-specific binding. MCs were stained with PE−anti-CD117, FITC−anti-FcεRI, and PECy7−anti-IL-33R (all Biolegend). To investigate the activation of p65/RelA in p65/RelA−eGFP MC/9 reporter MCs after stimulation, we determined the eGFP expression by flow cytometry. The cells were analyzed with an LSR II flow cytometer (BD), and the data were analyzed with FlowJo 10 (Treestar Inc., Ashland, OR, USA).

### 4.7. Lysis and Immunoblotting

After stimulation with rmIL-33 and/or rmSCF (both 50 ng/mL) (Peprotec), BMMCs were lysed in lysis buffer (20 mM HEPES, pH 7.5; 10 mM EGTA, 40 mM β−glycerophosphate, 2.5 mM MgCl_2_, 2 mM orthovanadate, 1 mM dithiothreitol, 20 µg/mL aprotinin, and 20 µg/mL leupeptin supplemented with 1% Triton). The protein concentration was determined (BCA−kit; Pierce), and the samples were boiled (with 6 × Laemmli buffer). The lysates were separated with 10% sodium dodecyl sulfate (SDS) −Laemmli gels and afterwards transferred onto nitrocellulose membranes (biostep) by Western blotting. The membranes were blocked (with dry milk) and incubated with anti-pT180/pY182−p38, anti-p38, anti-pS177/181−IKK2, anti-p184/187−pTAK1, anti-pT202/pY204−ERK1/2, anti-pS32−IκBα, anti-IκBα, anti-pS536−p65, anti-p65, HSP90, anti-pST183/Y185−JNK1/2, anti-JNK1/2, anti-pS727−STAT3, anti-STAT3, anti-pS235/236−S6, anti-S6, and anti-tubulin (all from Cell Signaling except anti-IKK1/2, anti-TAK1, and anti-ERK1/2 (Santa Cruz, CA, USA)). The membranes were washed with TBS/0.1% Tween and incubated with HRP-conjugated secondary anti-rabbit Ig, anti-mouse Ig, or anti-goat Ig (SeraCare). We used ECL reagent (Pierce) for detection.

### 4.8. ELISA

After stimulation (for 24 h) with rmIL-33 and/or rmSCF (both 50 ng/mL) (Peprotec)**,** supernatants were collected and analyzed for IL-6 or IL-13 by using matched paired antibodies (ebioscience). For the ELISA experiments, we used at least 3 biological replicates separated into at least five technical replicates.

### 4.9. Isolation of mRNA and qPCR

After stimulation with rmIL-33 and/or rmSCF (both 50 ng/mL) (Peprotec), total RNA was extracted with the Quick-RNA™ Miniprep Kit (Zymo Research, Irvine, CA, USA) according to the manufacturer’s instructions. The RNA was reverse transcribed using the Oligo(dt)18 primer and SuperScript™ IV Reverse Transcriptase (Thermo Fisher Scientific, Waltham, MA, USA) according to the manufacturer’s instructions. RT−qPCR was performed using template cDNA and primers for IL-13 (mouse IL-13 qPCR primer pair, Sino Biological, Beijing, China, MP200252), IL-6 (forward 5′−TCTCTGCAAGAGACTTCCATCCAGT−3′; reverse 5′−AGCCTCCGACTTGTGAAGTGGT−3′; synthesized by BioTeZ, Berlin, Germany), ribosomal protein S29 (forward 5′−GAAGTTCGGCCAGGGTTCC−3′; reverse 5′−GAAGCCTATGTCCTTCGCGT−3′; synthesized by BioTeZ), and actin (forward fw 5′−CCACAGCTGAGAGGGAAATC−3′; reverse 5′−TCTCCAGGGAGGAAGAGGAT−3′; synthesized by BioTeZ) as the housekeeping gene. The PowerUp SYBR Green Master Mix (Applied Biosystems, Waltham, MA, USA) was used for detection. The samples were analyzed in duplicate in the AB 7500 Real-Time PCR System (Applied Biosystems) according to the manufacturer’s instructions. The expression levels of IL-13 and IL-6 transcripts were normalized to the housekeeping genes.

### 4.10. Statistical Analysis

The experiments were performed at least three times with at least three biological replicates (bone marrow cultures from different mice). Thereby, every biological replicate consisted of the pooled bone marrow of 2 mice. For the cytokine ELISA experiments, biological replicates were split into at least 4 technical replicates. The significance was assessed by Student’s *t*−test (* *p* ≤ 0.05; ** *p* ≤ 0.01; *** *p* ≤ 0.001). Statistics were performed with SigmaPlot 14.5 (Systat Software Inc., Erkrath, Germany).

## Data Availability

The data presented in this study are available on request from the corresponding author.

## Supplementary Materials

The following supporting information can be downloaded at: https://www.mdpi.com/article/10.3390/ijms231810855/s1.
