# Supplementary material for: The Heat Shock Protein 90 (HSP90) Is Required for the IL-33-Induced Cytokine Production in Mast Cells (MCs)
Source: Int J Mol Sci. 2022 Sep 17;23(18):10855. doi: 10.3390/ijms231810855 (PMC9502846; doi:10.3390/ijms231810855)
Supplement: Supplementary file 1 [file ijms-23-10855-s001.zip › ijms-1898605-supplementary.pdf]

International Journal of
Molecular Sciences

MDPI

Supplementary Figures:

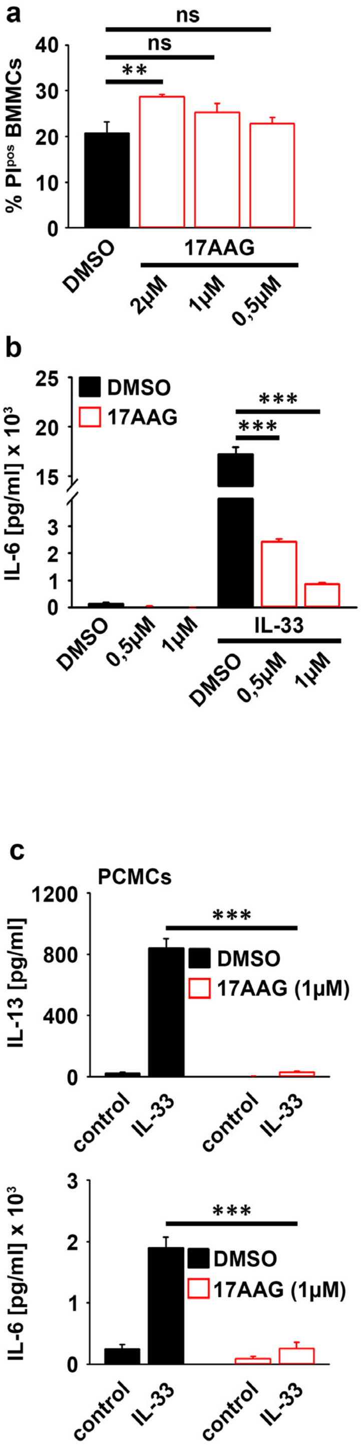

Figure S1. HSP90 mediates the IL-33-induced cytokine production. **(a)** BMMCs treated were with DMSO (vehicle) or 17AAG as indicated. Cells were analyzed by flow cytometry for PIpos cells (the summary of n=3 biological replicates is shown, unpaired students *t*-test; ns: not significant, ** $p \leq 0.01$). **(b)** BMMCs were treated with DMSO (vehicle) or 17AAG as indicated and were stimulated with IL-33 (50ng/ml) for 24h. Supernatants were collected and analyzed by ELISA (the summary of n=3 biological replicates is shown, unpaired students *t*-test; *** $p \leq 0.001$). **(c)** PCMCs were treated with DMSO (vehicle) or 17AAG (1µM) and were stimulated with IL-33 (50ng/ml) for 24h. Supernatants were collected and analyzed by ELISA (the summary of n=3 biological replicates is shown, unpaired students *t*-test; *** $p \leq 0.001$).

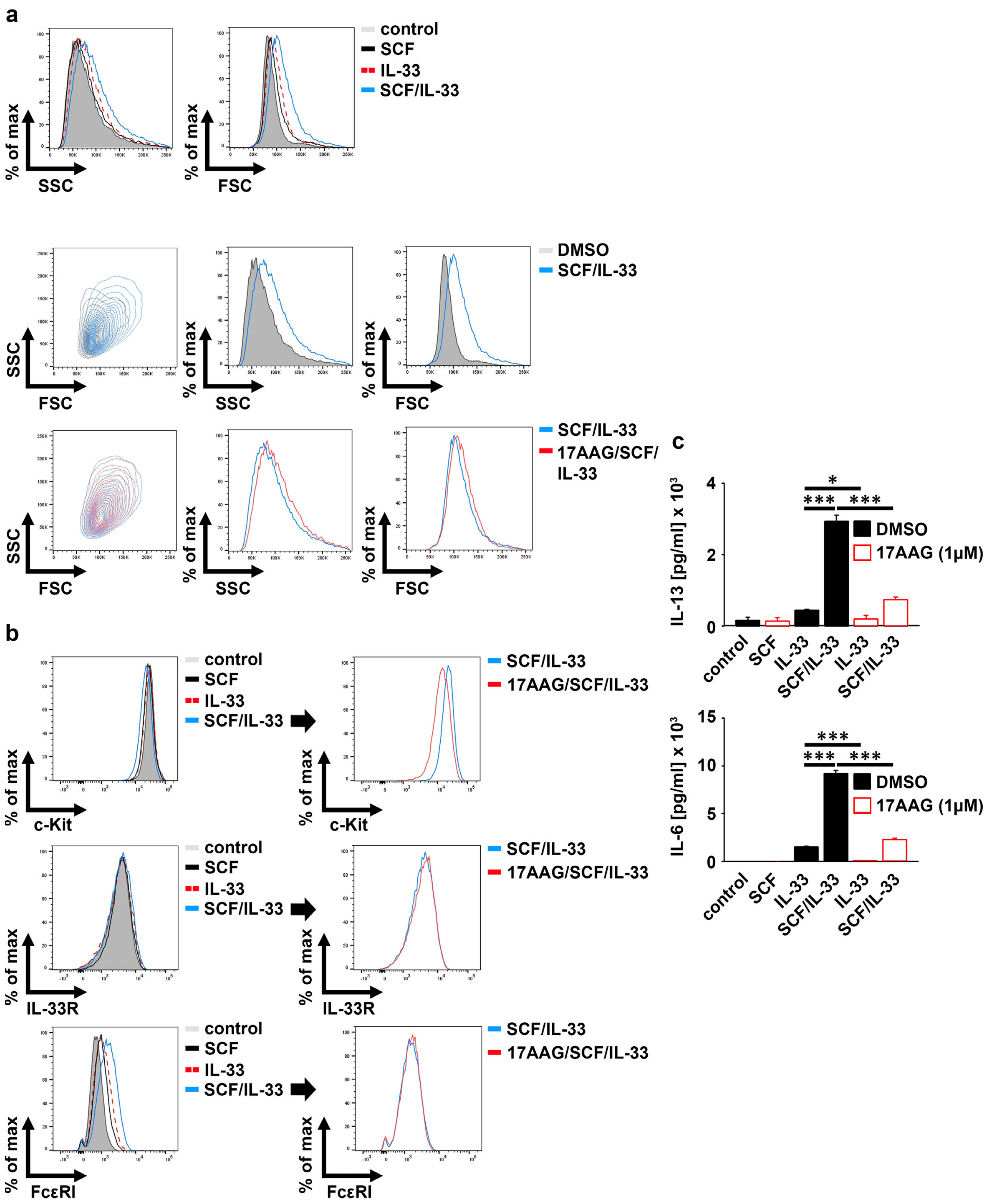

Figure S2. HSP90 mediates the SCF-supported and IL-33-induced cytokine production. **(a, b)** BMMCs treated with DMSO (vehicle) or 17AAG (1µM). Cells were stimulated with SCF or IL-33 or both together (both 50ng/ml) for 24h. Cells were analyzed by flow cytometry (representative are experiments is shown). **(c)** BMMCs were treated with DMSO (vehicle) or 17AAG (1µM) and were stimulated with SCF or IL-33 or both together (both 50ng/ml) for 24h. Supernatants were collected and analyzed by ELISA (the summary of n=3 biological replicates is shown, unpaired students *t*-test; * $p \leq 0.05$, *** $p \leq 0.001$).

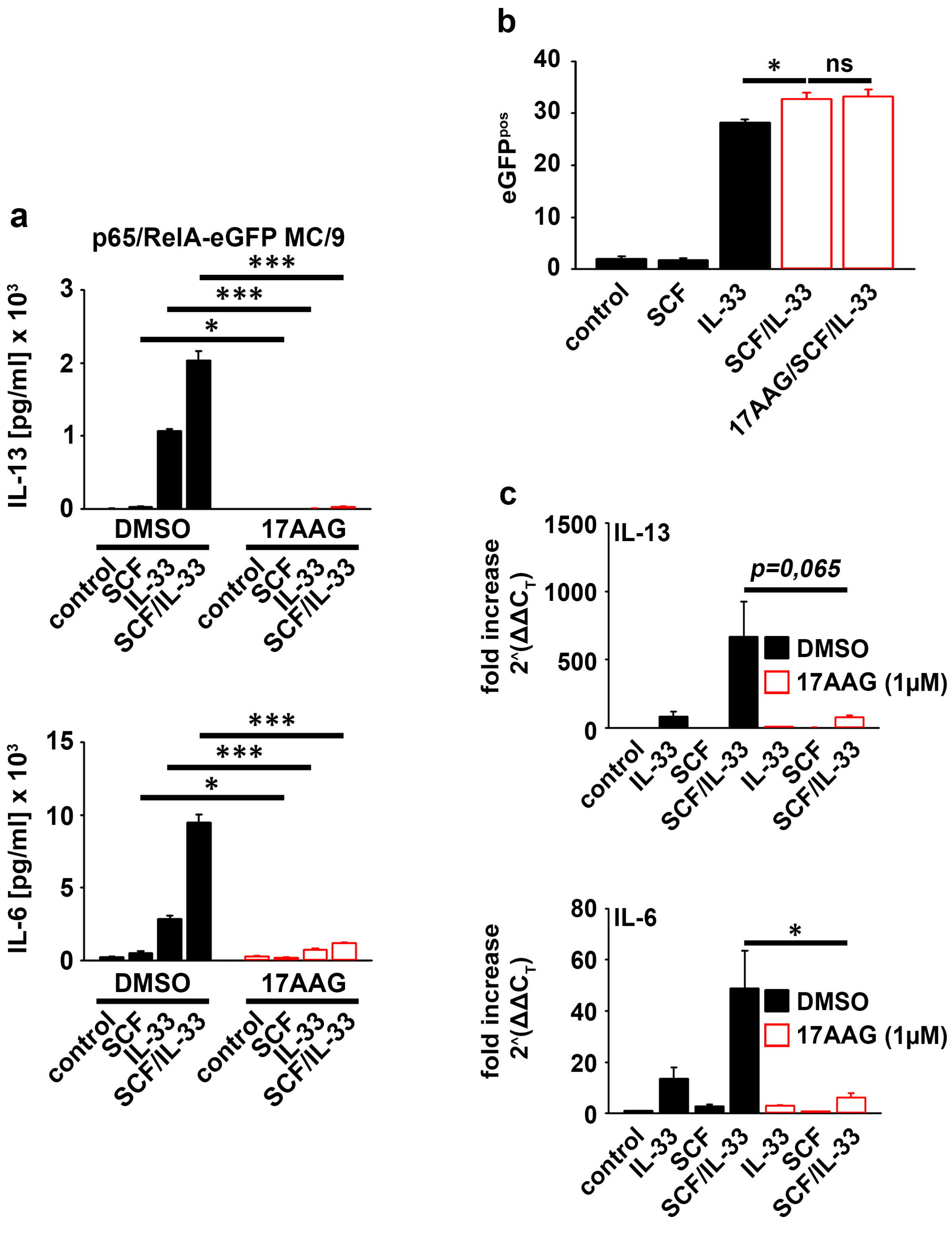

Figure S3. HSP90 mediates cytokine transcript stability. **(a)** P65/RelA-eGFP MCs were treated with DMSO (vehicle) or 17AAG (1μM). Afterwards, cells were stimulated with SCF or IL-33 or both together (both 50ng/ml) for 24h. Supernatants were collected and analyzed by ELISA (the summary of n=3 replicates is shown, unpaired students *t*-test; * $p \leq 0.05$, *** $p \leq 0.001$). **(b)** P65-eGFP MCs were treated with DMSO (vehicle) or 17AAG (1μM). Cells were stimulated with SCF or IL-33 or both together (both 50ng/ml) for 24h. Cells were analyzed by flow cytometry for eGFP expression (the summary of n=3 replicates is shown, ns not significant, unpaired students *t*-test; * $p \leq 0.05$). **(c)** BMMCs were treated with DMSO (vehicle) or 17AAG (1μM) and were stimulated with SCF or IL-33 or both together (50ng/ml) for 6h. Cells were harvested and analyzed by qPCR (the summary of n=3 biological replicates is shown, unpaired students *t*-test; * $p \leq 0.05$).
